# DN1p or the “Fluffy” Cerberus of Clock Outputs

**DOI:** 10.3389/fphys.2019.01540

**Published:** 2020-01-08

**Authors:** Angélique Lamaze, Ralf Stanewsky

**Affiliations:** Institut für Neuro und Verhaltensbiologie, Westfälische Wilhelms University, Münster, Germany

**Keywords:** *Drosophila melanogaster*, circadian clock, DN1p, locomotor activity, temperature response

## Abstract

*Drosophila melanogaster* is a powerful genetic model to study the circadian clock. Recently, three drosophilists received the Nobel Prize for their intensive past and current work on the molecular clockwork (Nobel Prize 2017). The *Drosophila* brain clock is composed of about 150 clock neurons distributed along the lateral and dorsal regions of the protocerebrum. These clock neurons control the timing of locomotor behaviors. In standard light–dark (LD) conditions (12–12 h and constant 25°C), flies present a bi-modal locomotor activity pattern controlled by the clock. Flies increase their movement just before the light-transitions, and these behaviors are therefore defined as anticipatory. Two neuronal oscillators control the morning and evening anticipation. Knowing that the molecular clock cycles in phase in all clock neurons in the brain in LD, how can we explain the presence of two behavioral activity peaks separated by 12 h? According to one model, the molecular clock cycles in phase in all clock neurons, but the neuronal activity cycles with a distinct phase in the morning and evening oscillators. An alternative model takes the environmental condition into consideration. One group of clock neurons, the dorso-posterior clock neurons DN1p, drive two peaks of locomotor activity in LD even though their neuronal activity cycles with the same phase (late night/early morning). Interestingly, the locomotor outputs they control differ in their sensitivity to light and temperature. Hence, they must drive outputs to different neuropil regions in the brain, which also receive different inputs. Since 2010 and the presentation of the first specific DN1p manipulations, many studies have been performed to understand the role of this group of neurons in controlling locomotor behaviors. Hence, we review what we know about this heterogeneous group of clock neurons and discuss the second model to explain how clock neurons that oscillate with the same phase can drive behaviors at different times of the day.

## Introduction

The fundamental function of the circadian clock is to synchronize the organism with its ecological niche. The circadian period is genetically determined (Konopka and Benzer, 1971) and therefore, does not depend on the environment, like the ambient temperature for example. However, 24 h oscillations of environmental parameters, such as daily light and temperature cycles (TC), synchronize the clock. Also, clock-controlled behavior is phased (time of occurrence within the 24 h period) based on the current environmental status. An individual’s locomotor activity pattern therefore depends on complex neuronal networks, integrating both environmental inputs and genetically encoded endogenous time information. *Drosophila melanogaster* is a reference model to study the circadian clock not only for its tremendous genetic advantages (Nobel prizes 2017), but it is also easy to tightly control the environment when it comes to study its locomotor behavior. When isolated in a small glass tube, the “dew lover” *Drosophila* displays a highly plastic locomotor behavior that changes with light and temperature. In standard light–dark 12–12 h (LD) cycles and constant mild temperature (22–25°C), male flies present two peaks of locomotor activity. In the late night, flies start to wake up in a synchronous manner and increase their locomotor activity. The light transition induces a startle response, after which the activity decays and male flies start their siesta. Then, in the late afternoon, they again increase their locomotion in a synchronous manner “anticipating” the lights-off transition. Because these activities occur several hours before the light-transitions, they have been defined as anticipatory. However, at cooler temperatures (18°C), the morning anticipation is strongly dampened or delayed, while the evening one is advanced compared to 25°C. Inversely, at warmer temperatures (29°C), the amplitude of the morning anticipation increases and advances while the evening anticipation delays (Majercak et al., 1999).

In constant light and temperature (LL), fruit flies are arrhythmic. This is due to the constitutive degradation of the clock protein TIMELESS (TIM), mediated by the circadian photoreceptor *cryptochrome* (*cry*) (Stanewsky et al., 1998). However, flies can entrain to TC in LL (LLTC 25–16°C) (Glaser and Stanewsky, 2005; Yoshii et al., 2005). Nonetheless, the behavior observed when only light alternates is different from the one observed in LLTC, which is again different from the one observed in the same TC but in constant darkness (DD) (Gentile et al., 2013). In LLTC, we observe a unique anticipatory activity peak at the end of the thermophase, while in DDTC this peak shifts toward the beginning of the thermophase.

**FIGURE 1:**
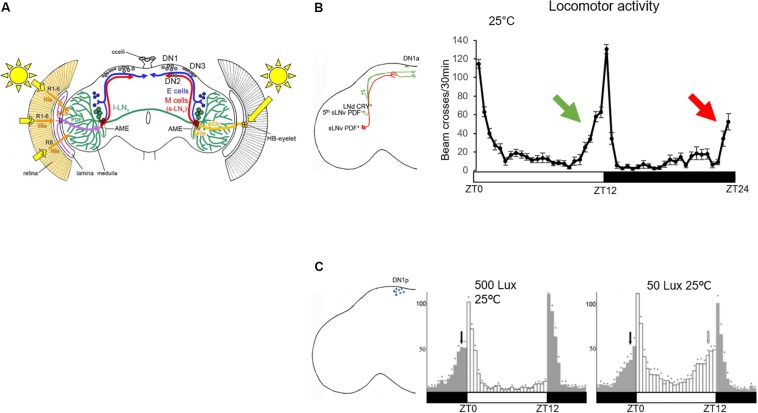
Role of clock neurons in controlling the locomotor behavior in standard LD conditions. **(A)** Clock neurons in the *Drosophila* brain. Figure taken from Helfrich-Förster (2019). Numbers 1–3 indicate the three putative opsin-based light input pathways to the clock neurons in addition to the HB-eyelet input (Helfrich-Förster, 2019). The l-LNv are represented in green, the LN-EO (E cells) in blue and the LN-MO (M cells) in red. The dorsal neurons (DN1–DN3) are colored in gray. Their projection pattern is not shown. The axonal projections of the lateral neurons are located in the dorso-posterior area of the brain (except for the lLNv, which project to the ipsi- and contralateral medulla). Most of the clock neuron dendritic projections are in the accessory medulla (AME) (Li et al., 2018). **(B)** Left: schematic representation of the LN-MO and LN-EO only, with their output projections. Right: locomotor activity trace of a group of 31 control flies (iso31) in standard LD and constant 25°C. The green arrow indicates the control of the evening anticipation by the EO (green cells in the brain) and the red arrow the morning anticipation by the MO (red cells in the brain). **(C)** Left: schematic representation of DN1p cell bodies in the brain; right: locomotor activity traces of *per^0^,w;Clk4.1M-Gal4/UAS-per16* (functional clock only in the DN1p) taken from Zhang Y. et al. (2010) in two different light conditions. White bars represents 12 h of light, dark bars 12 h of darkness.

The *Drosophila* brain clock is composed of about 150 clock neurons (Figure 1A). In 2004, two labs showed that if a functional clock is restricted to a group of CRY^+^ lateral neurons (LN) expressing the neuropeptide pigment dispersing factor (PDF), this is sufficient to drive the morning anticipation, which therefore was named lateral neurons-morning oscillator (LN-MO). In contrast, a clock restricted to LN expressing CRY but not PDF is sufficient to drive the evening anticipation (Figure 1B), and therefore was defined as lateral neurons-evening oscillator (LN-EO) (Grima et al., 2004; Stoleru et al., 2004).

In 2010, Zhang Y. et al. (2010) showed that a clock in a group of about 12 clock neurons located in the dorsal part of the protocerebrum, the DN1p, is sufficient to drive both morning and evening anticipation, albeit under distinct light and temperature conditions (Figure 1C). It was the first time that a group of clock neurons was found capable of controlling locomotor activity twice a day. By definition, a circadian output is a behavior that occurs every 24 h. Therefore, how do clock neurons with a 24-h molecular clock cycling in phase, control locomotor activity twice a day? Contrary to the EO and the MO whose neuronal activities cycle at different phase (Liang et al., 2016), the neuronal activity cycles in phase within the DN1p group (Flourakis et al., 2015; Liang et al., 2016). However, there is heterogeneity within this group. Half of them express *cry* (Benito et al., 2008), not all of them are glutamatergic (Hamasaka et al., 2007; Chatterjee et al., 2018) and a certain proportion of them express the neuropeptide DH31 (Kunst et al., 2014) or allatostatin-C (Diaz et al., 2019). It is still unclear which subgroup of DN1p controls the morning activity and which one controls the evening one, although the glutamatergic and CRY*^+^* DN1p seem to have a predominant role in regulating morning activity (Chatterjee et al., 2018).

To understand how DN1p clock neurons contribute to the complex circadian regulation of locomotor behavior, we review what is known about this intriguing cluster of neurons and will try to emphasize how their comprehension can help us to understand how neurons integrate and relay multiple inputs.

## DN1p: a Non-Autonomous “Circadian” Oscillator

**FIGURE 2:**
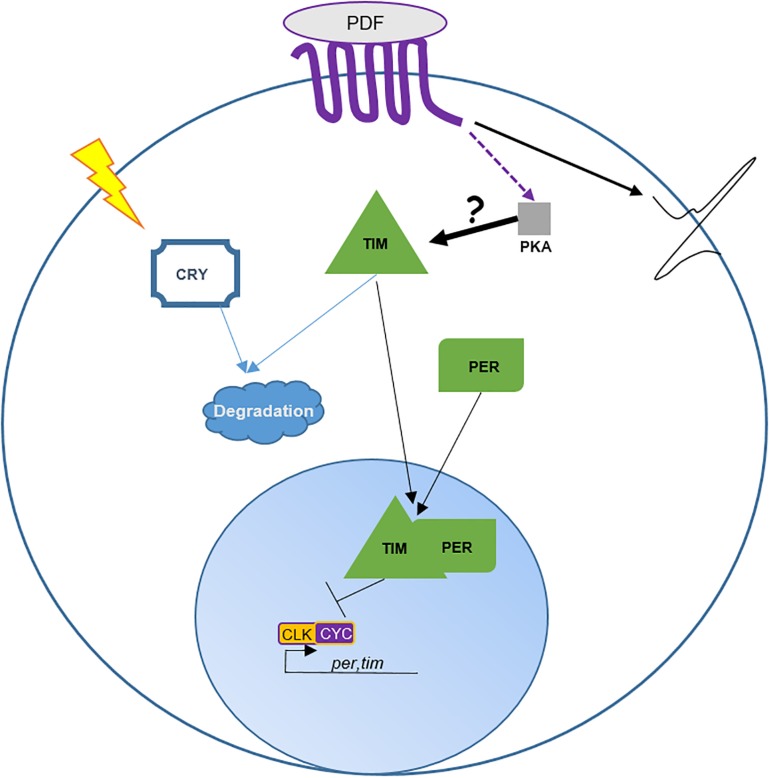
Role of PDF in DN1p entrainment and neuronal activity. This cartoon is based on Seluzicki et al. (2014). The transcription factors CLOCK (CLK) and CYCLE (CYC) form a heterodimer promoting the expression of *period* (*per*) and *timeless* (*tim*). PER and TIM proteins undergo various post-transcriptional and post-translational modifications delaying their nuclear translocation and inhibition of their own expression by binding to CLK/CYC (Tataroglu and Emery, 2015). *Pdfr* encodes for a seven transmembrane GPCR protein (Hyun et al., 2005). Activation of PDFR leads to an increase of cAMP in the cytoplasm. cAMP interacts with and activates PKA activity. Directly or indirectly PKA stabilizes TIM in the cytoplasm. In about half of the DN1p TIM is degraded by light via CRY. Interestingly, Seluzicki et al. (2014) observed that PDF promotes DN1p neuronal activity independently of PKA. Hence, the authors suggest that PDF cells entrain the DN1p molecular clock via the control of TIM by PKA and promote DN1p neuronal activity in a PKA-independent manner.

By definition, circadian clocks tick autonomously. In the absence of environmental input (DD 25°C), organisms maintain their rhythm of about 24 h, they free run. In the *Drosophila* brain, clock proteins maintain their rhythms for days in most of the clock neurons including the DN1p, while most of the peripheral clocks stop their oscillations after a few days in constant condition (Veleri et al., 2003). However, in the absence of PDF or in the absence of PDF cells, the DN1p loose their oscillations in DD very quickly (Veleri et al., 2003; Klarsfeld et al., 2004; Yoshii et al., 2009), suggesting a dependency of these neurons on the LN-MO. Indeed, when the pace of the molecular clock is genetically modified in the LN-MO, clock proteins and *tim* mRNA oscillate in the DN1p following the pace of the LN-MO (Stoleru et al., 2005; Chatterjee et al., 2018). How do PDF neurons dictate the rhythm to the DN1p? When PDF links to its receptor (PDFR, a G protein-coupled receptor), this leads to an increase of cAMP (Shafer et al., 2008), which activates protein kinase A (PKA). Interestingly, Seluzicki et al. (2014) have observed that a rescue of PER oscillations specifically in the LN-MO is sufficient to drive TIM oscillations in DN1p. They suggested that PDF regulates TIM levels via a PKA signaling pathway (Seluzicki et al., 2014). However, we do not know whether TIM is directly phosphorylated and stabilized by PKA in a PDF-dependent manner. Nonetheless, this hypothesis provides a nice model of the DN1p pace-regulation by the LN-MO (Figure 2). Furthermore, in the absence of both CRY and PDF, PER expression in the DN1p becomes arrhythmic even in LD (Cusumano et al., 2009), reinforcing this dependency toward the LN-MO.

Hence, although they express the molecular circadian machinery, the DN1p miss an unknown element providing autonomy, potentially a factor that regulates TIM oscillations in a CRY and PDF independent manner. Like peripheral clocks, the DN1p are not autonomous and only maintain their rhythm in DD thanks to the PDF^+^ pacemaker neurons.

## The Role of DN1p in Regulating Locomotor Behavior in Constant Darkness

**FIGURE 3:**
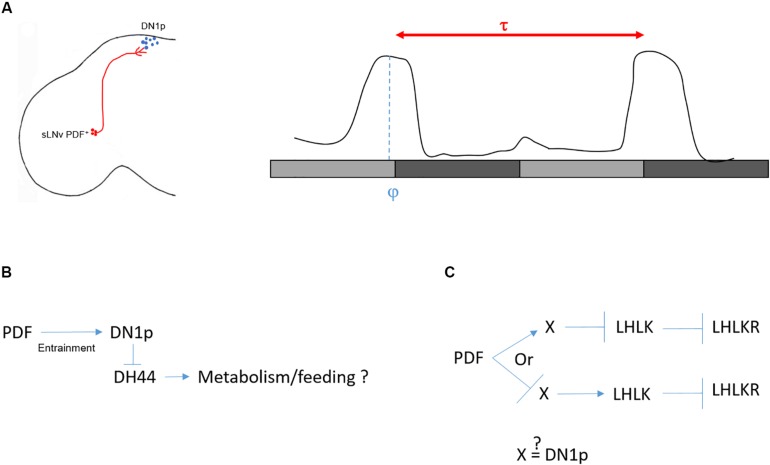
Control of locomotor activity in DD. **(A)** Left: sketch of sLNv and DN1p in the *Drosophila* brain. Right: a typical locomotor behavior in DD. Light gray bars represent the subjective day, dark gray bars represent the subjective night. Flies free run with an activity peak at the end of the subjective day. The period (τ) is defined as the distance between two peaks. τ is controlled by the sLNv (red), while the phase (φ) (when the peak of locomotor activity happens within τ) is controlled by the DN1p (blue). **(B)** Several arguments speak against a role of DH44 in controlling locomotor rhythms in DD (see text). However, the DH44 neurons appear to be rhythmic with a phase opposite to the DN1p (Cavey et al., 2016), and they modulate feeding behavior (Dus et al., 2015). From this, we can postulate that clock neurons influence feeding behavior via rhythmic inhibitory action on the DH44 neurons. **(C)** In the second model (Cavey et al., 2016), the LHLK neurons are indirectly inhibited by PDF. Hence, either PDF activates an unknown intermediate (X) which in return inhibits the LHLK neurons, or PDF inhibits X which activates LHLK neurons. Since activation of LHLK downstream neurons, LHLKR, changes the phase of the locomotor activity without changing the period of inactivity (Cavey et al., 2016), it is possible that the intermediate are the DN1p neurons.

A rhythmic circadian output is defined by its period and the phase of its peak and trough. The period of a circadian output is genetically determined. However, the phase is determined by the interaction of the animal with its environment. In DD and constant temperature, the main peak of locomotor activity occurs in the subjective evening. The rhythm of this activity pattern is driven by the PDF^+^ neurons (Grima et al., 2004; Stoleru et al., 2004; Figure 3A). Interestingly, when a functional clock is restricted to these neurons, the flies remain rhythmic but their peak of activity shifts toward the subjective morning (Grima et al., 2004). Recently, Chatterjee et al. (2018) were able to change the phase of the DD locomotor activity without affecting the period by genetically modulating the speed of the DN1p clock: phase advanced when the clock was sped up and delayed when the pace was slowed down. While the PDF neurons determine the pace, the DN1p determine the phase. Therefore, we can propose that in the absence of light and temperature oscillations, the endogenous period of the locomotor rhythm is provided by the PDF^+^ neurons and the phase by the non-autonomous DN1p oscillator (Figure 3A). Hence, it seems logical to propose that the DN1p are downstream of the PDF neurons.

The DD locomotor rhythm of *pdf* mutant flies, or flies lacking its receptor (*pdfr*^–^), is strongly dampened (Renn et al., 1999; Hyun et al., 2005). Interestingly, the rhythm strength can partially be restored when PDF reception is rescued only in DN1p neurons (Zhang L. et al., 2010). Nonetheless, flies are still rhythmic in DD when DN1p output is inhibited using the expression of tetanus toxin (Guo et al., 2016). Even more surprisingly, *gl60j* mutants that lack DN1p neurons are still rhythmic in DD (Helfrich-Förster et al., 2001; Klarsfeld et al., 2004), hence questioning a direct influence of the DN1p on DD rhythmicity.

Looking for downstream neurons important for DD rhythmicity, Cavanaugh et al. (2014) screened for neuronal drivers that lead to arrhythmic locomotor behavior when activated using the thermoreceptor dTrpA1. They identified 10 driver lines with the commonality of showing expression in neuroendocrine cells in the pars intercerebralis (PI), the fly functional homolog of the mammalian hypothalamus. They focused on a line (*kurs58*-Gal4) that is expressed in 16–18 cells in the PI. After a transcriptomic analysis of individual *kurs58* PI cells, they found that some of these neuroendocrine cells express the neuropeptide DH44 (six cells). DH44 is a diuretic hormone, the *Drosophila* homolog of the stress hormone corticotrophin releasing factor (CRF) (Cabrero et al., 2002) and downregulation of DH44 expression using a pan neuronal driver strongly decreased rhythmic behavior (Cavanaugh et al., 2014). However, using the same pan neuronal driver and RNAi line, another group was not able to replicate this result (Cavey et al., 2016). Also, specific activation of DH44 neurons with dTrpA1 using the dh44^*VT*^-gal4 line does not increase the proportion of arrhythmic flies (see Table 1 in Cavanaugh et al., 2014), raising doubts about the real implication of these neurons in regulating DD rhythms. Nonetheless, they do seem to respond to rhythmic input, since their neuronal activity oscillates in DD (Cavey et al., 2016). Interestingly, *kurs58*-Gal4 is also expressed in sifamide-expressing PI (SIFa) neurons, which are not overlapping with the DH44^+^ cells (Cavanaugh et al., 2014). The authors observed a decrease of DD rhythmicity in flies deprived of the SIFa neurons (SIFa > reaper) (Cavanaugh et al., 2014) consistent with the observation that pan-neuronal downregulation of SIFa expression strongly decreases rhythmicity in DD (Cavey et al., 2016). Finally, Cavanaugh et al. (2014) found a physical interaction between the DN1p and both the DH44^+^ and the SIFa^+^ PI cells. Since kurs58-Gal4 is expressed in 16–18 PI cells including the six DH44^+^ and four SIFa neurons, we cannot conclude with certainty the influence of the DH44 neurons on locomotor rhythmicity in DD. It would be interesting to further test the potential role of the SIFa neurons.

Later, the same group proposed a potential circuit downstream of the DH44 neurons responsible for DD rhythmicity (King et al., 2017). Flies express two receptors for DH44, DH44R1 and DH44R2 (Hector et al., 2009). While DH44R1 neurons are found in the central nervous system (Dus et al., 2015; King et al., 2017), DH44R2 expressing cells were found in the gut, potentially within the enteroendocrine cells (Dus et al., 2015). King et al. (2017) proposed that a group of DH44R1 neurons localized in the subesophageal zone (SEZ) and expressing the neuropeptide *hugin* are the downstream target of the DH44 neurons for regulating DD rhythms. However, neither the null mutant for *dh44r1* nor the expression of the *dh44r1* RNAi in hugin^+^ neurons lead to arrhythmicity in DD (see Supplementary Table 1 of King et al., 2017). Nonetheless, the authors observed arrhythmic locomotor activity in DD when hugin^+^ neurons, along with many others using the *R21A07* driver line, are activated (King et al., 2017). DH44 neurons project dendritic arborization to the dorsal region of the SEZ, their cell bodies are highly positive for the pre-synaptic marker syt-GFP, and they send axonal projections to the gut (Dus et al., 2015). No projections have been observed in the thoracic ganglion, where motor neurons receive inputs from the brain. This projection pattern, along with the absence of arrhythmic behavior in *dh44r1* mutants, is therefore not compatible with a role for DH44 neurons in regulating locomotor activity rhythms (Figure 3B).

Another study used a genetic approach to find the circuit downstream of the clock responsible for rhythmic behavior in DD (Cavey et al., 2016). They screened for peptidergic function via expressing RNAis pan-neuronally. From this screen, they found that downregulation of leukokinin (LK) leads to a high percentage of arrhythmic flies (65.4%). LK is expressed in only four non-clock neurons in the adult brain: two in the lateral horn (LHLK neurons) and two in the SEZ. Because of their location and their projections to the dorsal region of the brain, they focused on the LHLK neurons and measured their activity in constant conditions using GCaMP6. Activity of these neurons peaks at the end of the subjective day and reaches a minimum at the end of the subjective night. Interestingly, the LHLK neurons are indirectly inhibited by PDF, suggesting that PDFR^+^ neurons modulate the activity of these neurons (Cavey et al., 2016). However, we do not know whether the DN1p are directly connected with the LHLK neurons (Figure 3C). Finally, the authors confirmed the rhythmic activity of this circuit by recording the neuronal activity from neurons that express the leukokinin receptor and project to the proximal region of the LHLK neurons (LHLKR neurons). Interestingly, activation of LHLKR neurons using the R65C07 driver line, for 1 day in DD, changes the phase of locomotor activity. When these neurons are active, flies are constantly active during the subjective day but decrease their activity during the entire subjective night instead of showing a wild-type peak of activity at the end of the subjective day (Cavey et al., 2016). Hence, although the pattern of activity is different from controls and the phase is not restricted to the subjective evening, they are still rhythmic, suggesting that the LHLK circuit is downstream of the DN1p to phase locomotor behavior in DD. However, it is important to consider that the R65C07 driver is expressed in many other cells in the brain, and hence the behavior observed may not be driven by the LHLKR neurons.

In summary, in DD and constant temperature, the LN-MO relays the rhythmicity, while the DN1p shape and phase the locomotor activity toward the subjective evening (Figure 3A) but the downstream circuits remain ambiguous. How can we explain the specific implication of these two groups of neurons in the control of the locomotor rhythms in DD? There are non-clock cells that express PDFR (Im and Taghert, 2010), such as Ring neurons (R neurons) in the ellipsoid body (EB), a structure that belongs to the central complex, the integration center for locomotion (Young and Armstrong, 2010). Hence, the activity of some of these R neurons could be directly influenced by diffusible PDF and therefore their rhythm could be under direct control of the PDF neurons (Liang et al., 2019). While the sLNv pacemaker would control the pace of these R neurons via PDF, the DN1p could phase the same or other neurons that would belong to the central complex. Although the DN1p do not physically interact with R neurons in the EB, indirect connections exist (Guo et al., 2018; Lamaze et al., 2018a) (see final chapter: “Role of DN1p in Temperature-Dependent Sleep Regulation”).

## The Role of DN1p in Regulating Locomotor Activity in Light–Dark Cycles and Constant Mild Temperatures

Interestingly, light intensity and temperature modulate the evening output of the DN1p (Figure 1C) (Zhang Y. et al., 2010). At 25°C the DN1p-driven evening anticipation is only visible at low light intensity (≤50 Lux). However, when the temperature drops to 20°C we can observe this output also under higher light intensities (Zhang Y. et al., 2010). How does the environment influence these DN1p outputs?

While a clock in the PDF cells is not necessary for morning anticipation (Stoleru et al., 2004; Zhang Y. et al., 2010), PDF neuropeptide or the presence of the PDF cells are (Renn et al., 1999). Interestingly, rescuing *pdfr* only in the DN1p restores morning anticipation (Zhang L. et al., 2010). Clock proteins in the DN1p cycle with high amplitude in LD, even in the absence of either *Pdf* or *cry* (Cusumano et al., 2009). However, the fact that *Pdf* mutant flies do not show a morning anticipation suggests that the neuronal activity of the DN1p, and therefore their output, depends on the neuropeptide PDF. Indeed, a shot of PDF excites the DN1p in a PKA independent manner (Seluzicki et al., 2014). This suggests that in addition of maintaining a robust molecular rhythm, PDF promotes the morning anticipation output via modulating DN1p neuronal activity (Figure 2). The rhythmic neuronal activity of DN1p depends on the sodium leak channel *narrow abdomen* (*na*) (Nash et al., 2002; Flourakis et al., 2015). Remarkably, *na*^*har*^ mutants lose their morning anticipation and also, the startle response to light-on (Nash et al., 2002; Zhang L. et al., 2010). Both behaviors are restored when *na* expression is rescued in the DN1p (Zhang L. et al., 2010). In *gl^60*j*^* mutants, which lack all retinal photoreceptors and the DN1p, both morning anticipation and startle response are absent as well (Helfrich-Förster et al., 2001). This suggests that DN1p are necessary for the morning peak, but they need PDF at the end of the night to induce the increase of locomotion around dawn.

The second DN1p output regulates the evening anticipation. However, this output is highly sensitive to the surrounding environment, as for example, higher light intensities inhibit it (see above and Figure 1C). Interestingly, the evening peak does become visible at higher light intensity in the absence of PDF or when its expression is reduced by half (*pdf*^0^/+) (Chatterjee et al., 2018). Furthermore, the PDF level is influenced by light intensity, as its expression increases with light (Chatterjee et al., 2018). From a yeast one-hybrid screen for transcriptional regulators of *Pdf* the nuclear receptor *Hr38* was found as a potential candidate (Mezan et al., 2016). Knock down of *Hr38* in PDF neurons leads to a decrease of PDF levels (Mezan et al., 2016) and consequently, the DN1p evening output becomes visible under high light condition (Chatterjee et al., 2018).

Intriguingly, this opposite effect of the PDF on DN1p outputs is comparable to the temperature effect on the morning and evening peaks (Majercak et al., 1999). Historically, the first *pdfr* mutant described was named *han*, which means “cold” in Korean (Hyun et al., 2005). However, apart from this interesting coincidence, it is not known if PDF levels vary with temperature.

The DN1p show heterogeneity in their response to PDF. Some respond positively to PDF neuron activation, others are inhibited (Chatterjee et al., 2018). This could explain the antagonistic effect of PDF on DN1p outputs. The morning DN1p would be activated by PDF, while the evening DN1p would be inhibited (Chatterjee et al., 2018). How this antagonistic response is regulated is not known.

One possibility is that PDF reception differs between morning and evening DN1p. The *Pdfr* locus encodes four isoforms with different coding exons, notably, isoforms C and D have an extra C-terminal coding exon in the intracellular part of the receptor (FlyBase), suggesting distinctive functions downstream of PDF reception. The *Pdfr* mutant allele *han*^5304^ causes a deletion of all seven transmembrane domains (Hyun et al., 2005). Hence, it is unlikely that the mutated protein will localize at the cell surface. *han*^5304^ mutants show a behavioral phenotype equivalent to *Pdf* null flies: absence of morning anticipation, an advance of the evening peak in LD and arrhythmic behavior in DD. However, the mutant allele *han*^3369^, which leads to a partial deletion of the C terminal domain potentially affecting the C and D isoforms only, maintains its morning anticipation, while causing arrhythmicity in DD (Hyun et al., 2005; Im and Taghert, 2010). This suggests that PDF may differentially affect neurons depending on the specific *Pdfr* isoforms they express. However, the CRY^+^ vGlut^+^ DN1p are sufficient to drive morning anticipation in standard LD (Chatterjee et al., 2018) and PDFR-MYC, a construct faithfully reporting PDFR expression, is exclusively expressed in the CRY^+^ DN1p (Im and Taghert, 2010). Therefore, we propose that PDF promotes the activity of the CRY^+^ morning DN1p neurons, which in turn inhibit the activity of the evening DN1p.

## Role of DN1p in Temperature Entrainment

The brain clock can be entrained by light and temperature (Wheeler et al., 1993). While about half of the clock neurons in the brain expresses *cry* and therefore can be entrained by light in the absence of a functional visual system (Emery et al., 2000), they cannot be entrained by temperature in the absence of the periphery, and more specifically, the chordotonal organs and aristae (Sehadova et al., 2009; Yadlapalli et al., 2018). In *nocte* mutants, the chordotonal organs are structurally defective, and flies do not properly entrain to TC, while synchronization to light is normal (Glaser and Stanewsky, 2005; Sehadova et al., 2009; Chen et al., 2018). Wild-type flies can be entrained to TC even with a small temperature variation of only 2°C (Chen et al., 2015; Yoshii et al., 2005). The *Drosophila* ionotropic receptor IR25a is specifically required for synchronization to such low-amplitude TC (2°C) in LL or DD (Chen et al., 2015). Since IR25a mutants do not affect high-amplitude temperature entrainment, we can therefore propose that synchronization to low- and high-amplitude TC uses different molecular and potentially neuronal thermo-circuits. Interestingly, TIM oscillations respond differently to the absence of this ionotropic receptor depending on the light condition. In low amplitude LLTC, TIM peaks between ZT16 and ZT18 in all wild-type clock neurons analyzed in this study. In *IR25a* mutants, however, TIM is constantly low in the DN1p and DN2 and its oscillations are strongly disturbed in the PDF neurons, but are not, or only weakly affected in the LNd and DN3 (Chen et al., 2015), suggesting an IR25a independent mechanism for their entrainment to low amplitude TC in LL. In the same low amplitude TC during DD, TIM peaks at different phases in the clock neurons: At ZT16 in the LN, at ZT10 in the DN2, and between ZT10 and ZT16 in the DN1p and DN3. In *IR25a* mutants, TIM is at constitutively low levels in the LN, but oscillations remain unchanged in the DN2, DN3 and in the DN1p, although the amplitude is dampened and the peak narrowed. The DDTC situation is difficult to interpret because one could think that if clock neurons become insensitive to temperature entrainment they should maintain their oscillations and free run. Hence, we do not understand why TIM levels are low and flat in the LNd and sLNv. Nonetheless, in low-amplitude LLTC, the effect of *IR25a* on TIM expression in the DN1p and DN2 is very clear. Furthermore, when DN1p or DN2 neuronal activity is inhibited using tetanus toxin, flies fail to synchronize to a low amplitude TC in LL (Chen et al., 2015), also supporting a role for the DN1p (and DN2) in these conditions.

In the wild, the daily variation of temperature follows the sun. Hence, to test whether we can distinguish a light oscillator from a temperature one, like in plants (Michael et al., 2003), we need to uncouple light and TC. Using an environmental uncoupling protocol, where LD and TC were offset by 6 h, TIM expression in the lateral neurons followed the LD regime, while the dorsal neurons were preferentially entrained by temperature (Miyasako et al., 2007). Later, applying a protocol with exactly opposite LD and TC, only the CRY^–^ DN1p, DN2, and DN3 followed the TC (Yoshii et al., 2010). Using a different sensory conflict protocol and PER immunostaining (which is less sensitive to light inputs), Harper et al. (2016) observed that only the DN2 and DN3 follow the TC. However, in the absence of *cry*, PER oscillations in all clock neurons analyzed followed the TC, contrary to what was observed during opposite LD and TC, where the LN-MO maintained its phase in accordance with the LD cycle (Yoshii et al., 2010). Nonetheless, it is striking to observe that in *cry*^0^ flies, PER oscillates with the highest amplitude in DN1p and DN2, whether light and temperature oscillations are in phase or not (Harper et al., 2016).

The TRPA channel *pyrexia* (*pyx*) is specifically required for synchronization to cold TC (20–16°C) (Wolfgang et al., 2013). While *IR25a* is expressed in the chordotonal neurons (Chen et al., 2015), *pyx* is expressed in the cap cells of the chordotonal organs as well as in the peripheral nervous system (PNS) (Wolfgang et al., 2013; Roessingh et al., 2019). *pyx*^3^ mutants do not properly synchronize to cold TC in DD and free run instead (Roessingh et al., 2019). Interestingly, while PER oscillations in the sLNv of *pyx*^3^ mutants seem to free run during the shifted TC, they are dampened in the DN1p.

Although *pyx* and IR25a are both expressed in chordotonal organs, they play a role in specific TC conditions, and differently affect temperature synchronization of various clock neurons. Chordotonal organs are present all over the *Drosophila* body. However, it is unclear how the temperature information is transferred from the chordotonal organs to the clock neurons. The light conditions are also an important factor because the DN1p are differentially affected by the absence of IR25a in presence or absence of light.

In summary, the DN1p are sensitive to temperature entrainment when they do not receive oscillating light inputs. Their molecular synchronization is strongly affected when chordotonal function or structure are disturbed (Chen et al., 2015, 2018; Roessingh et al., 2019). In low amplitude TC, their neuronal activity is required for behavioral entrainment. However, about half of them express *cry* and in a temperature-light conflict paradigm, they are synchronized with the light (Harper et al., 2016).

## Role of the DN1p in Temperature-Dependent Sleep Regulation

Sleep is a fundamental physiological process regulated by both homeostatic and circadian mechanisms. Sleep need accumulates during wakefulness and is released during sleep. In DD, the clock phases sleep (Shaw et al., 2000). However, a nocturnal mouse can switch to diurnality when its environment changes (food access and temperature) suggesting that the environment, independent of its entrainment function, can phase sleep (van der Vinne et al., 2014).

All animals with a complex central nervous system sleep, though the sleep pattern differs between species (Cirelli and Tononi, 2008). *D. melanogaster* is considered crepuscular for chrono biologists but diurnal for sleep biologists. In standard LD conditions, flies are mostly active around light transitions, explaining the crepuscular classification. In between, they show sleep-like behaviors, and because night sleep lasts longer than day sleep (also called siesta), they have been classified as diurnal. It is today clear that day and night sleep are differently regulated in flies (Ishimoto et al., 2012) and of different quality (Van Alphen et al., 2013). Consequently, siesta and night sleep might use different neuronal circuits.

In standard LD conditions, mutants for the peptide DH31 sleep slightly more than controls (Kunst et al., 2014). Especially during the morning anticipation period at the end of the night, flies are sleepier in the absence of DH31 (Kunst et al., 2014). Interestingly, some of the DN1p express this neuropeptide, and their activation delays day sleep onset and decreases sleep at the second half of the night (Kunst et al., 2014). This suggests that the DH31^+^ DN1p play a role in wakefulness around the light-on transition. Since this behavioral phenotype was obtained using ectopic DN1p activation, the question is if, and under which conditions, these clock neurons promote arousal in the morning?

**FIGURE 4:**
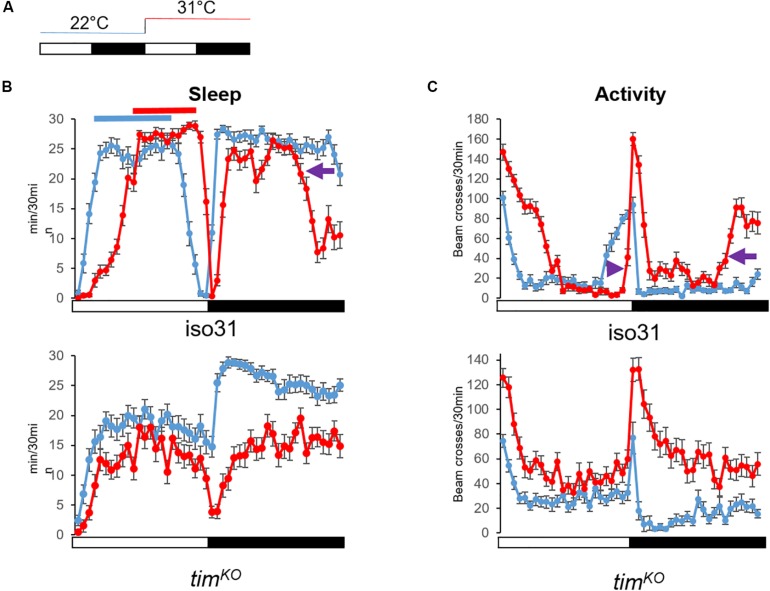
Warm temperature differentially affects day and night sleep. **(A)** Temperature protocol: White bar 12 h of light, dark bar 12 h of darkness. Flies were kept at 22°C the first day (blue line). The next day, temperature increased at light-on to 31°C for 24 h (red line). **(B)** Average sleep trace of control flies (iso31, *N* = 31) and clock mutant males [*tim*^*KO*^ (Lamaze et al., 2017), *N* = 32] (bottom graph). Sleep was measured as the sum of 5 min of inactivity per half hour. At warm temperature, wild-type flies delay their siesta. Both onset and offset are delayed (the red line shifts to the right compared to the blue line). At night and 31°C, flies sleep less. Notably, sleep offset is advanced compared to 22°C (purple arrow). On the other hand, the siesta of clock mutant flies is less affected, i.e., we do not observe a robust phase shift. Night sleep however is strongly dampened at 31°C. **(C)** Average locomotor activity of the same flies as in Graph **(B)**. The activity is measured as the sum of beam-crossings per half hour. We can observe the delay of the evening anticipation in iso31 flies (purple arrowhead), which corresponds to the delay of sleep offset. And while the morning anticipation is barely visible at 22°C (but clearer when looking at the sleep profile), at 31°C we can observe a clear advanced morning anticipation (purple arrow). Although clock mutant flies increase their locomotion in response to warm (startle response to light-on switches from 80 beam crosses/30 min at 22°C to 130 at 31°C), they do not present a phase shift of the trough of activity as observed in wild-type flies. At night, clock mutants maintain a high level of activity. Hence, we can see that the delay of the siesta at warm temperatures is clock dependent and independent of the acute response to temperature increase. Error bars represent standard error of the mean.

**FIGURE 5:**
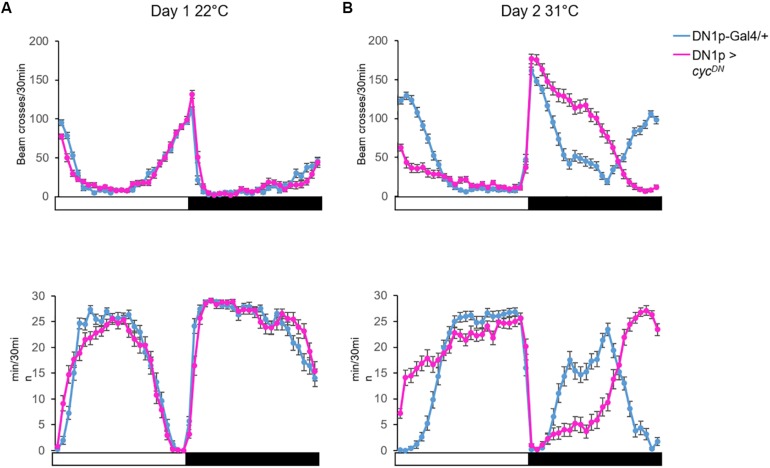
The DN1p clock is necessary to adapt locomotor behavior during warm temperatures. **(A)** Top: the locomotor activity monitored at 22°C on day 1; Bottom: the sleep trace in the same condition from the same flies. **(B)** Activity (Top) and sleep (Bottom) pattern of the same flies monitored the next day at 31°C. We compared the locomotor activity profile between control flies (*Clk4.1M*-Gal4/+) and flies with no functional clock in the DN1p (*Clk4.1M* > *cyc*^*DN*^)(Tanoue et al., 2004). *N* (*Clk4.1M*-Gal4/+) = 51; *N* (*Clk4.1M* > *cyc*^*DN*^) = 48; error bars represent standard error of the mean.

**FIGURE 6:**
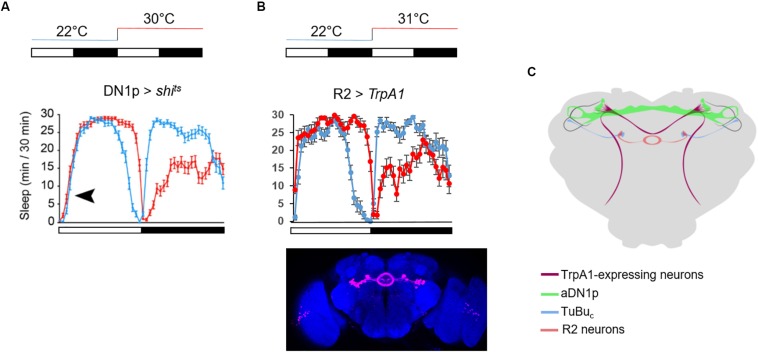
Timing the siesta onset during warm temperatures. **(A)** Average sleep trace of male flies expressing *shi*^*ts*^ in the DN1p. The temperature protocol is sketched above the graph. The sleep trace was copied from Lamaze et al. (2017) the black arrowhead indicates the absence of delay of the siesta onset at 30°C. **(B)** Average sleep trace of male flies expressing *TrpA1* in R2 neurons, using the same driver as in Lamaze et al. (2018a) (*R20D01*-Gal4). The brain picture of the expression pattern of R20D01 is a Z stack from the Janelia FlyLight database. TrpA1 is inactive at 22°C but active at 31°C. Note the remarkable symmetry between flies with inactivated DN1p and activated R2 neurons. *N* (*R2* > *TrpA1*) = 20. **(C)** Sketch of the neuronal circuit involved in timing the siesta based from Lamaze et al. (2017, 2018a, 2018b). The dorsal-projecting TrpA1^+^ neurons play an essential role in phasing the siesta with warm temperatures (Lamaze et al., 2017). They physically interact with DN1p clock neurons (Lamaze et al., 2017). The aDN1p directly inhibit a subset of TuBu_*c*_ neurons in the central domain of sAOTU (Lamaze et al., 2018a). These TuBu_*c*_ neurons promote sleep when activated at warm temperature. They interact in the superior region of the bulb with a subset of R2 neurons (Lamaze et al., 2018a).

An increase of temperature above 30°C phase-shifts the siesta, and both its onset and offset are delayed. These high temperatures also advance the offset of night sleep (Lamaze et al., 2017; Figures 4A,B). The delay of the siesta offset and the advance of the night sleep offset mirror the evening and morning anticipatory activity behaviors at warm temperature (≥27°C) (Majercak et al., 1999; Figure 4C). Interestingly, the delay of the siesta onset is also clock-dependent (Lamaze et al., 2018a). Hence, both the onset and the offset of the siesta phase-shift in a clock dependent manner (Figure 4B). Interestingly, inhibition of the DN1p neuronal activity at warm temperature (≥30°C), using *shibire*^*ts*^ (*shi*^*ts*^) (Dubnau et al., 2001), inhibits the delay of the siesta onset but not its offset (Lamaze et al., 2017; Figure 6A). This suggests that the DN1p promote morning arousal at warm temperature but their neuronal activity at the end of the day is not responsible for the phase delay of the siesta offset. Consistent with this, several groups have shown that the neuronal activity of the DN1p cycles along the day with a peak in the early morning (Flourakis et al., 2015; Liang et al., 2016) and a trough in the afternoon. Furthermore, stopping the clock in the DN1p via expressing a dominant negative form of *cyc* (Tanoue et al., 2004) does not affect the normal activity and sleep pattern at 22°C (Figure 5A), but their behavior becomes aberrant at 31°C (Figure 5B). Notably, during the day, the siesta is not restricted to a defined time of day. Interestingly, the night sleep is completely reversed compared to controls and the flies sleep when they should be awake and vice versa (Figure 5B). Therefore, the DN1p are essential for phasing behavior at temperatures ≥30°C, but at mild temperature, they do not play a fundamental role in controlling the locomotor pattern.

In contrast, Guo et al. (2016) have proposed that the DN1p promote sleep at mild temperatures, suggesting to have identified the first sleep-promoting clock neurons in *Drosophila*. They notably proposed that the DN1p promote sleep in the late day via inhibiting the LN-EO through a glutamatergic pathway (Guo et al., 2016). However, the LN-EO is sufficient to promote the increase of locomotion in the early evening before the light-off transition (Grima et al., 2004). Furthermore, live calcium imaging revealed that in standard LD the neuronal activity of the LNd oscillates in antiphase with the DN1p (Liang et al., 2016). Therefore, it is difficult to comprehend the relevance of an inhibitory action from DN1p sleep-promoting neurons on the LN-EO at a time when the DN1p are less active than the LN-EO. However, the locomotor behavior pattern changes with temperature, notably, at warm temperature (≥29°C) the evening anticipation is delayed compared to standard mild temperature (Majercak et al., 1999). Can the sleep-promoting DN1p inhibit the LNd in this condition and therefore delay the late-day wakefulness? This seems unlikely since the inhibition of the DN1p at ≥30°C does not inhibit the delay of the siesta offset (Lamaze et al., 2017; Figure 6A), suggesting that they are already inhibited at this time of day and temperature level. Finally, cooler temperatures (<20°C) advance the evening activity (Majercak et al., 1999), and the DN1p evening locomotor output is also promoted at cooler temperature (20°C) (Zhang Y. et al., 2010). Interestingly, Yadlapalli et al. (2018) have observed that cooling in the afternoon activated DN1p neuronal activity, suggesting again that the DN1p are pro-arousal. It would be interesting to test the role of a potential cooperation between LN-EO and DN1p to control the phase of the evening peak at cooler temperatures.

The thermoreceptor dTrpA1 phases the siesta and the morning anticipation at warm temperatures (>29°C) (Roessingh et al., 2015; Lamaze et al., 2017). Two groups of dTrpA1-expressing neurons, the *ppk* and the *dTrpA1[SH]* neurons, project to the dorso-posterior area of the brain where the DN1p, the LN-EO, and also the sLNv (LN-MO) project (Figure 6C). Downregulation of dTrpA1 expression in either the *ppk* neurons or the dTrpA1[SH] neurons, strongly dampened the phase shift of the siesta (both onset and offset) and the phase advance of the morning anticipation normally observed at warm temperature (≥30°C) (Lamaze et al., 2017). Since an inhibition of DN1p neuronal activity does not affect the delay of the siesta offset, we can propose that the dTrpA1-expressing neurons projecting to the dorso-posterior area of the brain, phase the siesta offset and onset in response to warm temperatures by directly interacting with the LN-EO and the DN1p, respectively.

The DN1p are a heterogeneous group, both transcriptomically and anatomically. Two sub-populations can be distinguished: one projecting anteriorly and terminating in the central domain of the small unit of the anterior optic tubercle (sAOTUc), and which are therefore called aDN1p (Figure 6C). The other group projects ventrally and posteriorly, and is therefore called vDN1p (Lamaze et al., 2018a). Guo et al. (2017) have described a driver line (JRC_SS00781 or Spl-DN1p) that restricts the number of sleep-promoting DN1p and their neurons belong exclusively to the vDN1p group (Lamaze et al., 2018a and Supplementary Figure 1 of Guo et al., 2017). Surprisingly, using an independent combination of driver lines to activate CRY^–^ DN1p, which do not project to the AOTU, the same lab found no sleep promotion (Guo et al., 2018), questioning the identity of the sleep promoting neurons.

The AOTU is a neuropil receiving visual input from specific neurons in the medulla (Omoto et al., 2017; Timaeus et al., 2017). At 31°C, activation of tubulo-bulbar neurons (TuBu_*c*_) that project dendrites to the sAOTUc inhibits the delay of the siesta onset but not the delay of the siesta offset, suggesting a sleep-promoting function. Importantly, DN1p inhibition at warm temperature (≥30°C) in the morning promotes activity of TuBu_*c*_ neurons, while activation of the DN1p in the warm afternoon inhibits the TuBu neuronal activity (Lamaze et al., 2018a). This suggests that the aDN1p promote arousal in the morning via the inhibition of their direct downstream target, the TuBu_*c*_ neurons. The TuBu neurons project to a microglomeruli structure called the lateral triangle (or bulb), where they interact with R neurons, which in turn project axons forming rings in the EB (Omoto et al., 2017; Timaeus et al., 2017). The sleep-promoting TuBu_*c*_ neurons interact with a subset of R2 neurons in the superior region of the bulb (Lamaze et al., 2018a; Figure 6C). Importantly, activation of these neurons at 31°C inhibits the delay of the siesta onset (Figure 6B), suggesting that TuBu_*c*_ neurons activate R2 neurons to promote sleep in the morning. Recently, Liang et al. (2019) measured rhythmic neuronal activity in different groups of R neurons including R2 (also called R4m). The EB is a neuropil structure integrating visual inputs and plays an important role in navigation (Sun et al., 2017). Hence, in order to fall asleep during daytime, this structure needs to decrease its sensitivity to visual inputs in order to increase the threshold of response. We can therefore propose that a subset of TuBu_*c*_ neurons that receive time information input from the DN1p promote sleep by decreasing the sensitivity of the EB to visual input via the activation of a subset of R2 neurons. During the night, flies do not receive any light input and activation of these R neurons does not inhibit the sleep loss induced by temperature (Figure 6B). *D. melanogaste*r is crepuscular. Hence, they are more active at dawn, especially at warm temperature. A subset of DN1p promote arousal during that time, according to the ambient temperature level.

## Conclusion and Future Directions

The brain clock controls sleep/wake rhythms. In *D. melanogaster*, only 150 neurons express clock genes and yet, we do not know how their time information is integrated by the central nervous system. The DN1p are a very peculiar group. First, their development correlates with the development of the visual system. *glass* encodes a transcription factor required for photoreceptor development (Moses et al., 1989). *gl^60*j*^* mutants have a profound defect of the visual system development and in addition, the DN1p fail to differentiate. This suggests a developmental and/or functional relationship between these clock neurons and the visual system (Helfrich-Förster et al., 2001; Klarsfeld et al., 2004). Second, although they express clock genes, the DN1p are not autonomous, and their DD rhythm entirely depends on the sLNv pacemaker neurons and the neuropeptide PDF (Klarsfeld et al., 2004). Finally, although the cells within this group are synchronized to the same phase (molecular clock but also presumably neuronal activity), the DN1p can drive both morning and evening activity under specific environmental conditions (Zhang Y. et al., 2010). This suggests a further separation within this group of at least two clusters.

The DN1p’s evening activity is inhibited by strong light (≥500 Lux) and is only visible at 25°C under low light condition (≤50 Lux). Interestingly, in DD the main peak of locomotor activity happens during the subjective evening. This phase is under the control of the DN1p (Chatterjee et al., 2018), suggesting that the DN1p that drive the evening activity under LD low light condition are the same as the ones phasing the locomotor activity in DD. Can we define the two clusters of DN1p more precisely? CRY is required to phase the onset of the siesta at warm temperatures (Lamaze et al., 2017) and the CRY^–^ DN1p do not project to the AOTU (Chatterjee et al., 2018; Guo et al., 2018). Hence, we can propose that the aDN1p, which promote wakefulness in the warm dawn, are CRY^+^. Furthermore, a clock in the vGlut^+^ DN1p is sufficient to drive the morning anticipation (Chatterjee et al., 2018), suggesting that the aDN1p are CRY^+^ and vGlut^+^. However, two of the CRY^+^ neurons are vGlut^–^ (Chatterjee et al., 2018), indicating that the DN1p-driven evening activity is under the control of a mix of CRY^+^ and CRY^–^ vGlut^–^ neurons.

The distinction of DN1p clusters is less clear when we focus on the projection to the PI. The DN1p physically interact with different neuroendocrine cells in the PI. Since the DH44 neurons do not send axonal projections to the thoracic ganglion, or to the central complex, and because downregulation of its receptor DH44R1 does not lead to arrhythmicity (King et al., 2017), it seems unlikely that these neuropeptidergic neurons influence locomotor rhythms in DD. However, this circuit could play a role in feeding rhythm and/or rhythmic metabolic processes (Dus et al., 2015; Figure 3B). The DH44 neurons are not the only neuroendocrine cells interacting with the DN1p (Barber et al., 2016; Cavey et al., 2016), and the DN1p are not the only clock neurons projecting to this region (Schubert et al., 2018). Hence, it would be of high interest to investigate the role of the clock neurons in the control of the PI-corpora cardiaca/corpora allata activity. This circuit is considered to be the functional homolog of the vertebrate hypothalamus–hypophysis circuit (Buch and Pankratz, 2009).

The central complex in *Drosophila* could be compared to the processor of a computer. It is the part of the brain where the computation happens in order to drive the appropriate behavior in response to the various inputs the brain receives. The central complex is composed of several neuropil structures, including the fan shaped body and the EB. A direct connection between the DN1p and neurons of the central complex is not known. However, the DN1p interact with cells that do connect to neurons projecting to the central complex. The aDN1p interact with a subset of TuBu_*c*_ neurons which in return contact a subset of R2 neurons in the superior part of the bulb. The symmetry of the behavior between flies with silenced DN1p and activated R2 neurons is striking (Lamaze et al., 2017; Figure 6B). Recently, Liang et al. (2019) have measured the neuronal activity of different groups of R neurons along 24 h during LD and the first day of DD. Interestingly, the group of R neurons that show the most homogeneous rhythmic activity are the R5 [confusingly called R2 in this study (Liang et al., 2019)]. We do not know however, whether activation or inhibition of R5 neurons affect the locomotor activity in DD, notably whether their inhibition would change the phase of the locomotor behavior in DD. Nonetheless, it is interesting to note that many neuronal groups in the brain display rhythmic activity (Barber et al., 2016; Cavey et al., 2016; Liang et al., 2019), and most of them do not interact directly with clock neurons. Actually, apart from the TuBu_*c*_ neurons (Lamaze et al., 2018a) and the PI neurons (Cavanaugh et al., 2014; Barber et al., 2016) which interact with the DN1p, none of the other groups directly interact with the DN1p. However, it is most likely that rhythmic signals end up in the central complex to control locomotor patterns. Interestingly, PDFR is expressed in different R neurons, including R2 and internal rings (Im and Taghert, 2010; Liang et al., 2019). Hence, it is totally conceivable that PDF neurons control the DD rhythm via secreted PDF action on the central complex, while the DN1p control the phase of the locomotor activity pattern via their indirect interaction with the EB.

To conclude, although the DN1p cannot be considered as a circadian oscillator, they play an essential role in phasing clock-controlled behaviors. Since the phase of a circadian behavior is the result of an integration of the environmental status with the time of day, this group of clock neurons seems to function at the crossroad between environmental input, in particular temperature, and the internal clock.

## Author Contributions

AL wrote the manuscript and RS commented on it.

## Conflict of Interest

The authors declare that the research was conducted in the absence of any commercial or financial relationships that could be construed as a potential conflict of interest.
